# Predicting the Unpredictable: Development and External Validation of the GO SOAR Surgical Risk Calculator for Data-Driven Predictions of Surgical Complications in Gynaecological Oncology Surgery

**DOI:** 10.3390/cancers18183058

**Published:** 2026-09-21

**Authors:** Faiza Gaba, Oleg Blyuss, Janna G. Oganezova, Giulia Pellecchia, Stefano Restaino, Cristian Dell’Acqua, Fabio Martinelli, Naia Seminario, Eloi Sirvent, Martina Aida Angeles, Antonio Gil-Moreno, Alexandra Nyiro, Sani Wong, Elly Brockbank, Eleanor Brierley, Sarah Wintle, Jyoti Utkar, Suzanne Rae, Mahalakshmi Gurumurthy, Michael Kirkham, Andrew Kerr, Gemma Owens, Bethany Pidd, Charlotte Bowles, Lucia Lo Cascio, Tamara Čopi, Andrej Cokan, Burak Giray, Çağatay Taşkıran, Dogan Vatansever, Shant Apelian

**Affiliations:** 1 Department of Gynaecological Oncology, University Hospital Southampton NHS Foundation Trust, Southampton SO16 6YD, UK; 2 Institute of Applied Health Sciences, University of Aberdeen, Aberdeen AB25 2ZD, UK; 3 Centre for Cancer Screening, Prevention and Early Diagnosis, Wolfson Institute of Population Health, Queen Mary University of London, London E1 4NS, UK; 4 Department of Pediatrics and Pediatric Infectious Diseases, Institute of Child’s Health, Sechenov First Moscow State Medical University, Sechenov University, 119435 Moscow, Russia; 5 Academician A.P. Nesterov Department of Ophthalmology, Institute of Clinical Medicine, Pirogov Russian National Research Medical University, 117437 Moscow, Russia; 6 Azienda Sanitaria Universitaria Friuli Centrale, 33100 Udine, Italy; 7 Department of Biomedical Sciences, Humanitas University, Via Rita Levi Montalcini 4, Pieve Emanuele, 20072 Milan, Italy; 8 Department of Gynecological Oncology-Humanitas San Pio X, Via Francesco Nava 31, 20159 Milan, Italy; 9 Gynecologic Oncology Unit, Vall d’Hebron Barcelona Hospital Campus, Universitat Autònoma de Barcelona, 08035 Barcelona, Spain; 10 Barts Health NHS Trust, London E1 2ES, UK; 11 University College London Hospitals NHS Foundation Trust, London NW1 2PG, UK; 12 Aberdeen Royal Infirmary, Aberdeen AB25 2ZN, UK; 13 Lancashire Teaching Hospitals NHS Foundation Trust, Preston PR2 9HT, UK; 14 University Hospital of Maribor, 2000 Maribor, Slovenia; 15 American Hospital, 34365 Istanbul, Turkey; 16 Department of Obstetrics, Gynecology & Reproductive Sciences, Yale School of Medicine, New Haven, CT 06510, USA

**Keywords:** surgical risk calculator, machine learning, surgical morbidity, surgical mortality, gynaecological oncology

## Abstract

**Background/Objectives**: Universal surgical risk calculators are not validated for use and poorly predict postoperative morbidity and mortality for women undergoing gynaecological oncology surgery. This adversely affects communication of risk resulting in poorly informed decisions and missed opportunities for medical optimization to mitigate risk preoperatively. We present the development and external validation of our novel GO SOAR surgical risk calculator for use to preoperatively predict postoperative thirty-day surgical morbidity and mortality in relation to gynaecological oncology surgeries. **Methods**: New logistic regression models were developed using the GO SOAR1 training cohort (n = 1811) and externally validated in an independent prospective cohort (n = 416) for two outcomes: thirty-day postoperative mortality (alive versus dead) and thirty-day postoperative morbidity (any complication (Clavien–Dindo I–V) versus none). Performance of the GO SOAR models was compared against established all-purpose surgical risk calculators (SORT/POSSUM/P-POSSUM/NSQIP). Model discrimination was assessed with sensitivity calculated at a clinically significant prespecified specificity threshold of 90%. **Results**: For mortality, AUROC was 0.752 (95% CI 0.570–0.935) for GO SOAR full and 0.795 (95% CI 0.660–0.930) for GO SOAR condensed; corresponding sensitivities at 90% specificity were 57.1% and 42.9%. For morbidity, AUROC was 0.698 (95% CI 0.641–0.755) and 0.703 (95% CI 0.646–0.760) for the full and condensed models, respectively, with sensitivities of 30.4% and 32.1% at 90% specificity. **Conclusions**: The GO SOAR model using a data-driven, gynaecological-oncology-specific approach at 90% specificity, achieved the highest observed sensitivity among the evaluated calculators. Accurate surgical risk predictions are crucial for major oncological surgery, where complications can diminish quality of life and affect long-term cancer survival. A model such as the GO SOAR surgical risk calculator that uses readily available preoperative data, regardless of income setting, is essential in reducing global disparities in surgical outcomes.

## Simple Summary

Accurate preoperative surgical risk predictions help predict potential postoperative complications, guide shared doctor–patient decision making, and personalise management strategies to improve perioperative surgical outcomes. We present the development and external validation of the novel GO SOAR surgical risk calculator using global prospective data across all income healthcare settings. At a clinically significant 90% specificity threshold, it achieved the highest observed sensitivity among the evaluated all-purpose universal surgical risk calculators commonly used as part of routine clinical practice, and is easy to use, minimizing intensive data entry and physiological parameters and laboratory tests, ensuring benefits to women globally irrespective of resource availability.

## 1. Introduction

Surgical risk prediction is central to patient–doctor shared decision making and facilitates transparent communication, leads to a reduction in uncertainty, safer surgeries, and better outcomes [1]. Studies have shown that clinicians are poor at predicting medical and surgical risk, and often rely on their experience, resulting in a subjective global assessment of patient fitness for surgery [2]. Surgical risk calculators are a set of objective tools providing quantitative assessments of predicted postoperative outcomes mitigating the highly variable subjective clinician-led perception of patient risk [2]. Common surgical risk calculators in clinical use to predict postoperative morbidity and mortality in gynaecological oncology surgery include the American College of Surgeons NSQIP (National Surgical Quality Improvement Program) [3] surgical risk calculator, POSSUM (Physiologic and Operative Severity Score for the Study of Mortality and Morbidity), P-POSSUM (Portsmouth Physiologic and Operative Severity Score for the Study of Mortality and Morbidity) [4], and SORT (Surgical Outcome Risk Tool) [5]. These universal all-procedure risk calculators have a multitude of weaknesses. All are developed from heterogeneous data, and so lack applicability to surgical subspecialties such as gynaecological oncology [6]. All-procedure surgical risk calculators such as NSQIP, POSSUM, and P-POSSUM require extensive data collection, limiting practicality. POSSUM and P-POSSUM overemphasize physiological tests, some of which are costly or not routinely performed, excluding their use in resource-poor settings where the burden of surgical morbidity and mortality is higher [7,8]. In addition, all-procedure calculators do not account for the wide range in complexity of gynaecological oncology surgeries or frailty of oncology patients with risk calculators such as SORT oversimplifying clinical complexity [6,9]. These challenges highlight a gap between the current use of all-procedure risk calculators and their ideal use in gynaecological oncology. They also highlight a need to create risk calculators that more aptly fulfil clinical needs in gynaecological oncology.

The GO SOAR Surgical Risk Calculator is a novel machine learning-based tool to predict thirty-day postoperative morbidity and mortality risk exclusively in women undergoing gynaecological oncology surgery. It has been developed using prospective data from six continents collected from the GO SOAR database [8]. In the present study, we used the GO SOAR1 development cohort to construct updated mortality and morbidity models with revised outcome definitions and predictor coding, and subsequently assessed these prespecified models in a separate prospective validation cohort. The present study therefore represents model redevelopment followed by external validation, rather than external validation of an unchanged previously published model. We additionally compared the performance of the updated GO SOAR models with commonly used all-purpose surgical risk calculators.

## 2. Materials and Methods

### 2.1. Source Data and Study Design

This study comprises two phases: model development using the GO SOAR1 prospective training cohort, followed by external validation in an independent prospective cohort. The GO SOAR1 training dataset included 1811 patients undergoing elective and emergency curative or palliative surgeries for primary or recurrent gynaecological malignancies across seventy-three hospitals in twenty-seven countries. Candidate predictors were selected a priori from three domains: patient, disease, and surgery, and are described in full in the previously published methodology [10]. Variables were restricted to those systematically available before surgery and obtainable without additional tests, ensuring applicability across all income settings. Sixteen candidate predictors were selected a priori to be included and processed. These were selected from three domains: patient, disease, and surgical predictors. Patient predictors included the following: age (linear); ethnicity (white versus non-white); body mass index (kg/m^2^, linear); haemoglobin (g/dL, linear); white cell count (109/L, linear); albumin (g/L, linear); American Society of Anaesthesiologists (ASA) grade (1–2 versus 3–5); and Eastern Cooperative Oncology Group (ECOG) performance status (0–2 versus 3–4). Disease predictors included the following: primary cancer (ovary, uterine, cervical, vulva/vagina); radiological FIGO stage (stage I–II versus stage III–IV); and neoadjuvant chemotherapy (yes versus no). Surgical predictors included the following: history of previous abdominal surgery (minimal access (laparoscopy/robotic) versus laparotomy); mechanical bowel preparation (yes versus no); intra-operative antibiotics (yes versus no); surgical modality (minimal access versus laparotomy); and surgical complexity score (estimated preoperatively based on radiological imaging). The surgical complexity score was divided into five separate groups: pelvic, bowel, urological, upper abdominal, and lymphadenectomy surgery. Each of these five groups was further subdivided into specific surgical procedures and allocated a complexity score based on expert consensus (Supplementary Table S1) [10]. The prediction time point was immediately before surgery. Surgical complexity and surgical modality were coded from the planned operative procedure, and these predictor values were therefore available before the operation commenced.

For external validation, additional prospective data were collected from oncology centres in high- and low–middle-income countries. Investigators included consecutive patients undergoing surgery for ovary, uterus, cervix, vulva, vagina, and gestational trophoblastic cancers over a thirty-day period (January 2025–February 2026). Inclusion criteria were identical to those for the training cohort [8]. Investigators were required to monitor patients for a minimum of thirty days postoperatively. Postoperative follow-up was dependent on local clinical pathways (in person, telephone, or review of medical records).

### 2.2. Model Development and Algorithm Selection

Two binary outcomes were modelled: thirty-day postoperative mortality (alive versus dead) and thirty-day postoperative morbidity (any complication, Clavien–Dindo I–V, versus none). For each outcome, a full model incorporating all candidate predictor variables was first fitted using the GO SOAR1 development cohort. A condensed model was then derived using stepwise selection based on Akaike’s Information Criterion (AIC) to identify the most parsimonious set of predictors without meaningful loss of model fit.

Body mass index and white cell count were re-specified as categorical variables to improve clinical interpretability and generalisability. Body mass index was categorised using World Health Organization standard thresholds (<25, 25–30, >30 kg/m^2^; reference category < 25 kg/m^2^). White cell count was categorised using data-derived tertiles from the training cohort (<6.10, 6.10–8.10, >8.10 × 10^9^/L; reference category < 6.10 × 10^9^/L).

To determine the most appropriate modelling approach, five candidate methods were compared on the training dataset using repeated stratified five-fold cross-validation (ten repeats, yielding fifty AUROC estimates per method): logistic regression (LR), support vector machines (SVMs), random forest, gradient boosting, and XGBoost. All methods were trained on an identical feature set. Discrimination was assessed using the area under the receiver operating characteristic curve (AUROC). Results are presented in Table 1 and Table 2. Logistic regression was selected as the final modelling approach on the basis of equivalent or superior discriminatory performance relative to more complex methods, together with its advantages of interpretability, transparency of coefficients, and suitability for deployment as a clinical tool across varied resource settings.

For mortality, the condensed model retained seven predictors: age, ECOG performance status, preoperative haemoglobin, body mass index category, neoadjuvant chemotherapy, FIGO stage, and surgical complexity score. For morbidity, the condensed model retained eleven predictors: surgical complexity score (moderate and high categories), surgical modality, FIGO stage, ASA grade, previous laparoscopy, previous laparotomy, neoadjuvant chemotherapy, mechanical bowel preparation, age, intra-operative antibiotics, and white cell count category.

### 2.3. External Validation

Before evaluation of the external validation cohort, the predictor coding, transformations, regression coefficients, and specifications of both the full and condensed models were fixed using the GO SOAR1 development cohort. The external validation cohort was used solely to evaluate the performance of these prespecified models and was not used for model selection, refitting or recalibration. Both full and condensed logistic regression models, developed on the GO SOAR1 training cohort, were applied to the external validation cohort to generate individualised predicted probabilities. The details of the GO SOAR1 cohort derived from 73 hospitals in 27 countries (41% data from low and middle income countries, 59% high income countries) has been previously published [8]. Model discrimination was assessed using the AUROC, and with sensitivity calculated at a clinically prespecified specificity threshold of 90%. In addition to AUROC, we report sensitivity at 90% specificity because this represents a clinically relevant operating point that limits false positives while maximising the detection of high-risk patients [11]. In a surgical setting where access to prehabilitation services to optimize surgical fitness is expensive and limited, maintaining high specificity is important before recommending further evaluation. A threshold of 90% has been adopted in validation studies across multiple medical disciplines, for example, cancer screening, medical imaging and biomarker studies [12,13,14,15].

Model calibration was assessed by comparing predicted versus observed event rates across risk deciles. Performance of the GO SOAR full and condensed models was compared against established all-purpose surgical risk calculators (SORT, POSSUM, P-POSSUM, NSQIP) applied to the same validation cohort.

### 2.4. Statistical Analysis

Discriminatory performance was quantified using AUROC with 95% confidence intervals. Pairwise differences in AUROC between GO SOAR and comparator models were assessed using paired DeLong tests. At the prespecified operating point of approximately 90% specificity, sensitivity, specificity, positive predictive value (PPV), and negative predictive value (NPV) were calculated for each model. Differences in sensitivity between models at this operating point were quantified using paired-bootstrap 95% confidence intervals. Pairwise comparisons were considered exploratory, and nominal *p*-values were interpreted in the context of multiple comparisons.

After data cleaning, there were no missing values in the candidate predictors used for model fitting in either the development cohort (n = 1811) or the external validation cohort (n = 416); therefore, no imputation was performed. Supplementary Table S2 summarises patient characteristics and events of the external validation cohort. Patient characteristics and events of the developmental cohort have been previously published [10]. The final GO SOAR models were logistic regression models fitted by maximum likelihood and had no tuning hyperparameters. Predictor coding and preprocessing rules were defined using the development cohort and fixed before application to the external validation cohort.

For the internal model-development comparisons, repeated stratified five-fold cross-validation with ten repeats was used. Predictor coding and the model specification were fixed using the development cohort and were not re-derived within individual cross-validation folds. The cross-validation analyses were used to compare candidate modelling approaches during model development, whereas performance in the independent external validation cohort provides the primary out-of-sample assessment of the final GO SOAR models.

For cross-validation comparisons during model development, 95% CIs were derived from the 2.5th and 97.5th percentiles of the distribution of AUROC estimates across cross-validation folds. Model calibration was further quantified using the observed-to-expected (O/E) ratio, calibration-in-the-large (CITL), calibration slope, and Brier score. Mean miscalibration was considered statistically significant where the 95% confidence interval of the CITL excluded zero. All statistical analyses were performed using Python 3.8 and R 3.5.1.

## 3. Results

### Model Development: Algorithm Selection

Table 1 and Table 2 present the internal cross-validation performance of five candidate machine learning methods for thirty-day mortality and morbidity prediction respectively, using the full feature set identical to the final logistic regression models.

For mortality prediction (24 events; 1.3% event rate), confidence intervals overlapped substantially across all five methods, and no method demonstrated a clinically meaningful improvement in discrimination over logistic regression (AUROC 0.702). Support vector machines (AUROC 0.710) and random forest (AUROC 0.712) showed near-identical performance. Gradient boosting (AUROC 0.663) and XGBoost (AUROC 0.661) produced lower mean AUROCs, though confidence intervals remained overlapping across all methods, reflecting the inherent instability of AUROC estimation with only 24 events.

For morbidity prediction (501 events; 27.7% event rate), logistic regression (AUROC 0.689) again performed equivalently to or better than the alternative methods. Random forest achieved an AUROC of 0.694—a difference of 0.005 with almost entirely overlapping confidence intervals—a margin that is not clinically meaningful. Support vector machines (AUROC 0.564) was the only method with non-overlapping confidence intervals relative to logistic regression. Gradient boosting (AUROC 0.678) and XGBoost (AUROC 0.683) produced lower mean AUROCs with overlapping confidence intervals.

On the basis of these results, logistic regression was selected as the modelling approach for both outcomes. This choice is supported by equivalent or superior discriminatory performance relative to more complex methods, and by the practical advantages of logistic regression in a clinical context: coefficients are directly interpretable, the model is transparent for regulatory and clinical governance purposes, and it is straightforwardly deployable as an online calculator without dependency on proprietary software.

Data were collected from 416 patients from ten hospitals across six countries (United Kingdom, Italy, Spain, Greece, Slovenia, Turkey). In total there were 78 (18.8%) minor complications (Clavien–Dindo I–II), 34 (8.2%) major complications (Clavien–Dindo III–V), and 7 (1.7%) deaths.

**Figure 1:**
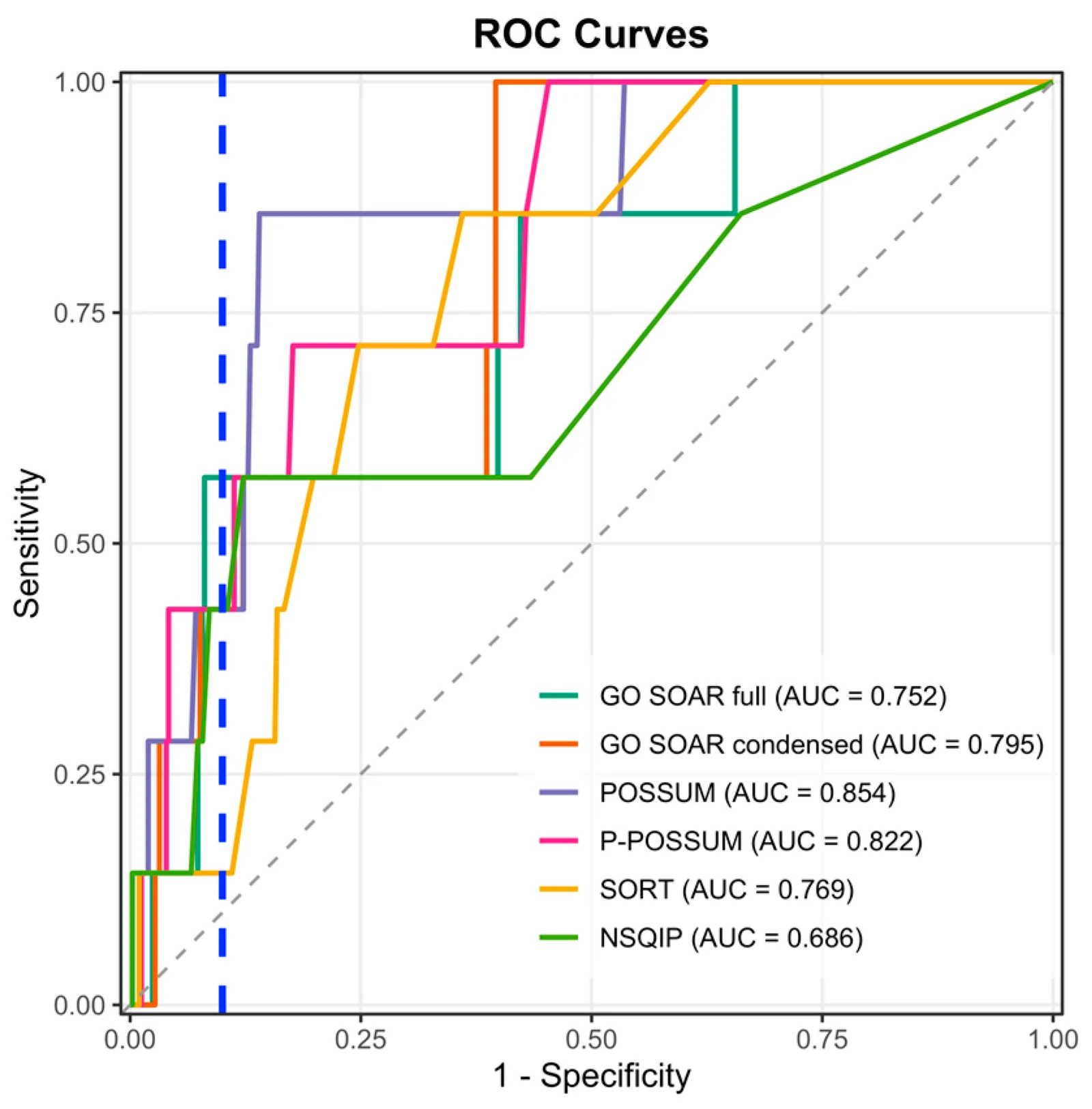
AUROC for thirty-day postoperative mortality prediction calculators.

Figure 1 compares the area under the receiver operating curve (AUROC) of all-purpose mortality calculators (SORT, NSQIP, POSSUM, P-POSSUM) and the GO SOAR (full-all fifteen variables, condensed–seven variables) mortality calculator. Table 3 summarises the mortality prediction sensitivity of each model at 90% specificity.

Figure 1 shows NSQIP to be the model with the poorest discrimination for mortality prediction, while SORT, POSSUM, P-POSSUM, and GO SOAR calculators have a similar average performance across all specificity thresholds. Table 3 summarises overall discrimination and performance at the prespecified operating point of approximately 90% specificity, including AUROC with 95% confidence intervals, sensitivity with 95% confidence intervals, specificity, PPV and NPV. AUROC estimates ranged from 0.686 (95% CI 0.446–0.927) for NSQIP to 0.854 (95% CI 0.720–0.988) for POSSUM. However, not all specificity thresholds are clinically significant, and at the prespecified 90% specificity threshold, the GO SOAR full model achieved the highest observed sensitivity of 57.1%, compared with 42.9% for POSSUM, P-POSSUM and NSQIP and 14.3% for SORT. PPV ranged from 2.5% to 10.8%, whereas NPV ranged from 98.4% to 99.2%.

Pairwise DeLong comparisons showed no robust evidence of an overall AUROC advantage for GO SOAR full over the comparator calculators (Table S2). The nominal comparison with NSQIP yielded *p* = 0.049 but did not remain significant after correction for multiple comparisons. Similarly, paired-bootstrap 95% confidence intervals for differences in sensitivity at the approximately 90% specificity operating point included zero (Table S3), indicating substantial uncertainty around the observed between-model differences.

**Figure 2:**
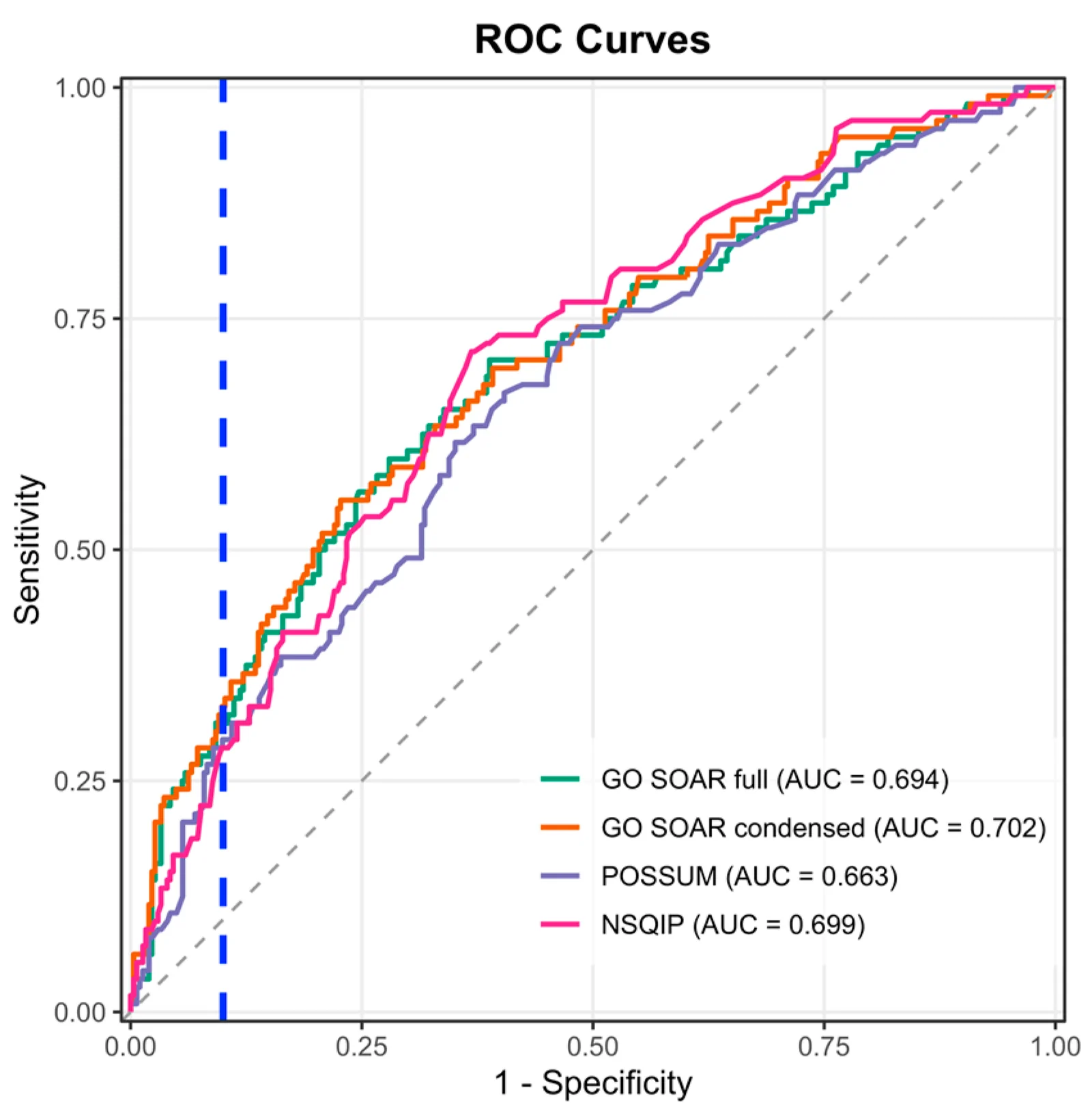
AUROC for thirty-day postoperative morbidity prediction calculators.

Figure 2 compares the AUROC of all-purpose morbidity calculators (NSQIP, POSSUM) and the GO SOAR (full-all sixteen variables, condensed–eleven variables) morbidity calculator. Table 4 summarises the morbidity prediction sensitivity of each model at 90% specificity.

Figure 2 shows POSSUM to be the model with the poorest discrimination performance for morbidity prediction, while the NSQIP and GO SOAR calculators have a similar average performance across all specificity thresholds. Table 4 summarises overall discrimination and performance at the prespecified operating point of 90% specificity, including AUROC with 95% confidence intervals, sensitivity with 95% confidence intervals, PPV and NPV. AUROC estimates were similar across all four calculators, ranging from 0.663 (95% CI 0.610–0.728) for POSSUM to 0.702 (95% CI 0.645–0.760) for GO SOAR condensed. At the prespecified 90% specificity threshold, the GO SOAR condensed model achieved the highest observed sensitivity of 32.1%, compared with 30.4% for GO SOAR full, 28.6% for POSSUM and 27.7% for NSQIP. PPV ranged from 51.6% to 54.5%, and NPV from 77.4% to 78.3%. Pairwise DeLong comparisons showed no statistically significant differences in AUROC between GO SOAR full and any comparator (Table S4). Similarly, paired-bootstrap 95% confidence intervals for the differences in sensitivity at the approximately 90% specificity operating point all included zero (Tables S5 and S6), indicating substantial uncertainty around the observed between-model differences.

**Figure 3:**
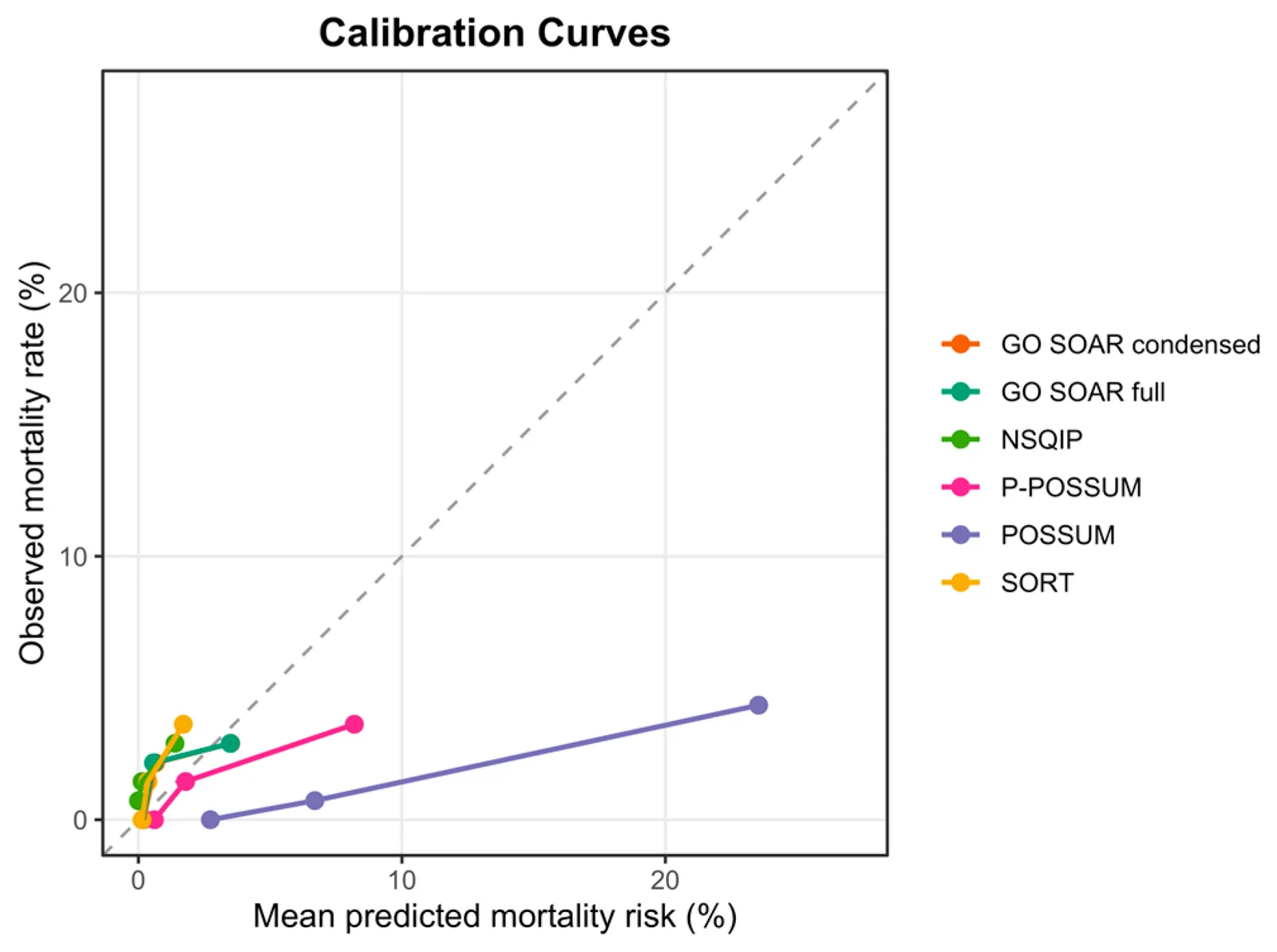
Calibration curves for thirty-day postoperative mortality prediction calculators.

**Figure 4:**
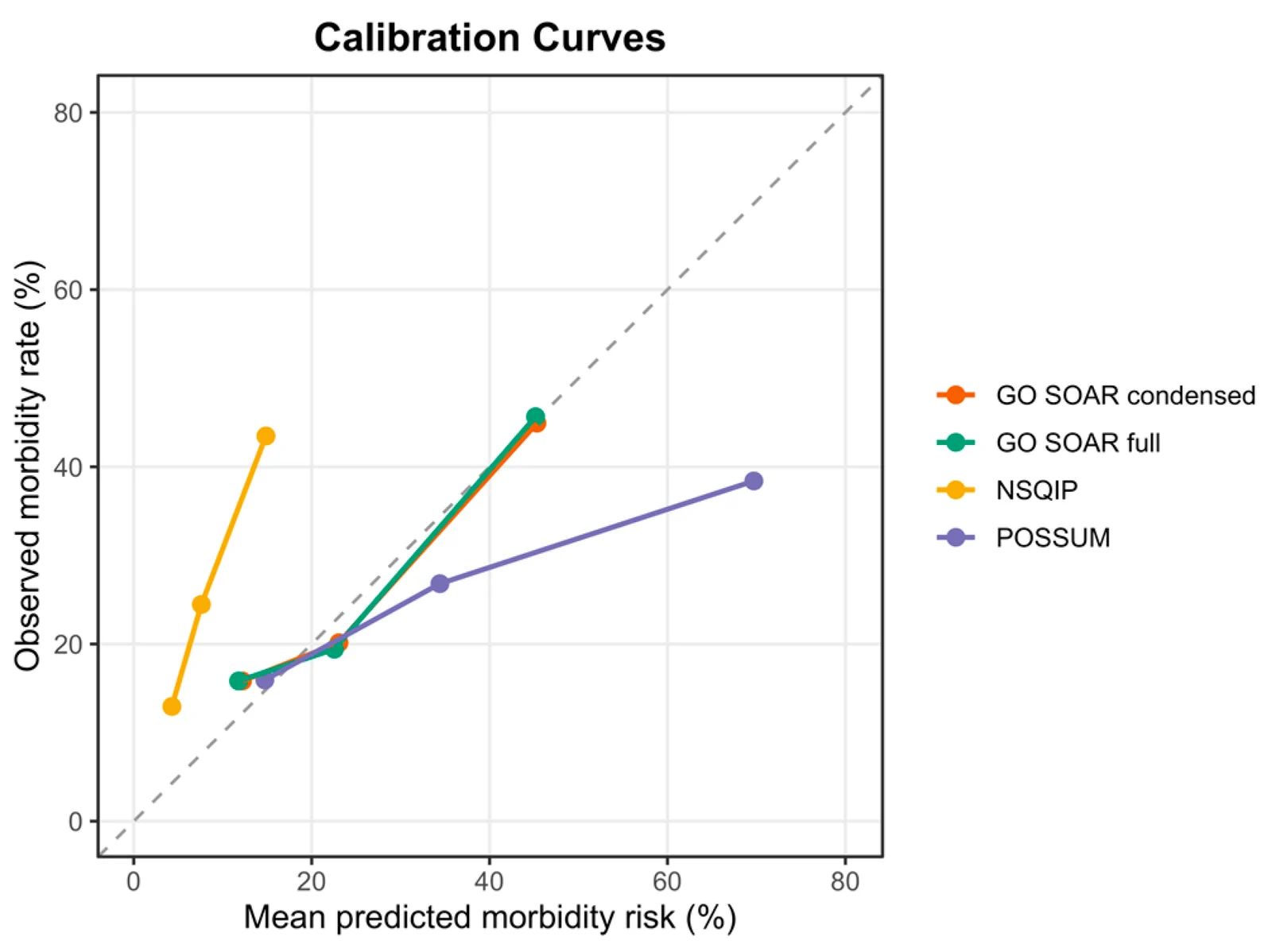
Calibration curves for thirty-day postoperative morbidity prediction calculators.

Figure 3 and Figure 4 compare the calibration for mortality and morbidity calculators respectively.

The mortality calibration curves show SORT and NSQIP to underpredict mortality, and P-POSSUM and POSSUM to overpredict mortality risk. The GO SOAR (full and condensed) model was the closest fitting model most accurately predicting mortality risk. The morbidity calibration curves show NSQIP to underpredict and POSSUM to overpredict morbidity and GO SOAR (full and condensed) showed closed agreement with the observed morbidity risk.

Table 5 and Table 6 present quantitative calibration statistics for all mortality and morbidity prediction models respectively, including observed-to-expected (O/E) ratio, calibration-in-the-large (CITL), calibration slope, and Brier score. Calibration-in-the-large was considered statistically significant where the 95% confidence interval excluded zero [16].

For thirty-day mortality (7 events, 1.7% event rate; Table 5), both GO SOAR models demonstrated the closest overall calibration to observed outcomes. The GO SOAR full and condensed models predicted 5.8 and 6.0 deaths respectively against 7 observed (O/E 1.20 and 1.17), with 95% confidence intervals spanning unity, indicating no statistically significant mean miscalibration. CITL values of 0.20 and 0.17 confirm near-perfect mean calibration, with confidence intervals that include zero for both models. In contrast, POSSUM substantially overpredicted mortality, yielding an expected total of 45.5 deaths against 7 observed (O/E 0.15; 95% CI 0.062–0.317), with a CITL of −2.16 (95% CI −3.018 to −1.471) entirely excluding zero, indicating highly significant and clinically dangerous overprediction. P-POSSUM also overpredicted mortality (E = 14.6; O/E 0.48; 95% CI 0.193–0.987). Conversely, SORT (O/E 2.30; CITL 0.86, 95% CI 0.003–1.543) and NSQIP (O/E 3.33; CITL 1.29, 95% CI 0.414–2.001) both significantly underpredicted mortality, with NSQIP additionally showing a calibration slope of 0.20, indicating that its predicted probabilities were compressed into a very narrow range with almost no spread across patients.

For thirty-day morbidity (112 events, 26.9% event rate; Table 6), the GO SOAR models demonstrated good calibration. The GO SOAR condensed model showed close agreement between predicted and observed risk, with an O/E ratio of 1.004 (95% CI 0.827–1.208), a CITL of 0.006 (95% CI −0.229 to 0.235), and a calibration slope of 0.936 (95% CI 0.655–1.232), with all confidence intervals including the values corresponding to ideal calibration (O/E = 1, CITL = 0, slope = 1). The GO SOAR full model performed similarly (O/E 1.018; CITL 0.027; slope 0.886). POSSUM significantly overpredicted morbidity, yielding an expected total of 164 complications against 112 observed (O/E 0.683; 95% CI 0.562–0.822), with a CITL of −0.768 (95% CI −1.023 to −0.520) entirely excluding zero, confirming systematic overprediction. The calibration slope of 0.464 indicates that POSSUM’s predictions are too extreme, assigning excessively high probabilities to high-risk patients and insufficient probabilities to low-risk patients. NSQIP substantially underpredicted morbidity (E = 37.0; O/E 3.025; CITL 1.396, 95% CI 1.168–1.618), with a calibration slope of 1.243, indicating that while its direction of risk ordering has some validity, its absolute predicted probabilities are generally too low for use in gynaecological oncology patients.

## 4. Discussion

In this study, we developed and externally validated a gynaecology-specific surgical risk prediction model specifically for predicting thirty-day postoperative morbidity and mortality for women undergoing major gynaecological oncology surgery. In the independent prospective external validation cohort, GO SOAR demonstrated encouraging discrimination and calibration and achieved the highest observed sensitivity among the commonly used in clinical practice calculators (SORT, NSQIP, POSSUM, P-POSSUM), at the prespecified 90% specificity operating point. For mortality, the small number of observed deaths results in limited precision around the performance estimates and between-model comparisons, and these results should therefore be interpreted accordingly. We show that our GO SOAR surgical risk calculator at the clinically significant 90% specificity threshold, outperforms common mortality and morbidity all-purpose surgical risk calculators (SORT, NSQIP, POSSUM, P-POSSUM) that are commonly in use in clinical practice. For both mortality and morbidity, pairwise statistical comparisons of AUROC and of sensitivity at 90% specificity did not reach conventional statistical significance, and these observed differences should therefore be interpreted as compatible with broadly equivalent discrimination between the GO SOAR and comparator calculators (Tables S2–S5). Our model is globally applicable and consists of variables that are readily available across all resource income settings. Our GO SOAR model is derived and validated in a global dataset.

Our results are in keeping with already published data showing that all-purpose multi-specialty surgical risk calculators (SORT, NSQIP, POSSUM, P-POSSUM) perform poorly and inaccurately predict risk within a gynaecological oncology cohort [17,18,19,20]. Reasons for poor performance are multifactorial. First, a universal calculator that incorporates multiple and heterogeneous surgical specialties necessitates that the degree of precision of the calculator must decrease. Specialty specific risk calculators have a better performance for predicting specialty specific surgical risks but are not generalizable [21,22,23]. Second, universal calculators such as NSQIP perform better for surgeries related to benign pathology suggesting that that there may be oncological processes affecting patient frailty that are too complex for the calculator to compensate for [17]. Third, universal calculators are unable to account for the variety of complex surgical procedures performed in gynaecological oncology which often involve multi-visceral organ resections in the pelvis and abdomen and occasionally chest/groins. Our GO SOAR surgical risk calculator accounts for this by including a surgical complexity score which accurately captures the full extent of complex operations with a comprehensive combined score across five surgical groups.

There exist multiple barriers to the use of universal surgical risk calculators in low-resource clinical settings. Calculators such as NSQIP are data intensive and rely upon the acquisition of detailed data points which are practically difficult to acquire in high-workflow environments with poor access to medical records. POSSUM and P-POSSUM are laboratory heavy-risk calculators and include specialist tests such as blood urea nitrogen (BUN). The preoperative laboratory results, collected as part of our international prospective GO SOAR database, indicate that this is not a widely performed test in resource-poor healthcare facilities in low–middle-income countries. In addition there exist validation gaps, with datasets used to validate universal risk calculators derived from high-income country settings and are not representative of low–middle-income country populations with different nutrition levels, disease patterns, or surgical indications.

Strengths of our GO SOAR calculator include its robust methodology, derivation and validation using a large dataset across both high- and low-resource healthcare settings, ease of use avoiding labour-intensive data entry and avoiding use of specialized physiological and laboratory tests, and the use of a surgical complexity score capable of accurately capturing complex gynaecological oncology surgeries.

Limitations include the small number of deaths in the dataset that was used to validate our model (24 deaths in development and 7 deaths in external validation), resulting in wide uncertainty around discrimination, sensitivity and calibration estimates. The GO SOAR database is continuing to prospectively capture surgical outcome data globally, and future collected data will be used to refine our model and improve predictions.

While surgical risk calculators are to be used as an adjunct to clinical decision making, they are not intended to be used in isolation and should not replace the advice of healthcare professionals about the potential risks or benefits of a planned surgical procedure. They do not predict long-term complications or quality of life. This is particularly important in gynaecological oncology where over the past decade there has been a push to increase the radicality of cytoreduction surgeries, for example, in ovarian cancer and gynaecological cancer recurrences. There must always be a careful discussion between the surgeon and patient discussing quality of life and survivorship versus complications as part of informed consent, with ultimately the patient deciding what trade-off between oncological survival and long-term survivorship quality of life is acceptable to them.

## 5. Conclusions

Our GO SOAR surgical risk calculator uses a data-driven approach to outperform at a clinically significant 90% specificity threshold, all-purpose universal surgical risk calculators which for too long have been providing inaccurate risk predictions for women with gynaecological malignancies undergoing complex surgeries. Our novel surgical risk calculator has been validated for use in clinical practice across all resource healthcare settings. It is designed to be used as an adjunct to clinical decision making to improve the communication of risk to patients preoperatively and better target resources to optimize medical fitness ahead of major gynaecological oncology surgery.

## Figures and Tables

**Table 1:** Internal cross-validation performance—30-day mortality (n = 1811, 24 events).

Method	Mean AUROC	95% CI	Performance vs. LR
Logistic regression	0.702	0.385–0.928	Reference
SVM (RBF kernel)	0.710	0.437–0.937	Overlapping CI; no meaningful difference
Random forest	0.712	0.343–0.909	Overlapping CI; no meaningful difference
Gradient boosting	0.663	0.422–0.806	Lower AUROC; Overlapping CI
XGBoost	0.661	0.400–0.844	Lower AUROC; Overlapping CI

AUROC: area under the receiver operating characteristic curve. CI: 95% confidence interval derived from the 2.5th and 97.5th percentiles across 50 cross-validation estimates (5-fold × 10 repeats). No formal significance test was applied; comparisons are based on overlap of confidence intervals and clinical relevance of AUROC differences. All models trained using identical features. Highlighted row = reference model selected for external validation.

**Table 2:** Internal cross-validation performance—30-day any morbidity (n = 1811, 501 events).

Method	Mean AUROC	95% CI	Performance vs. LR
Logistic regression	0.689	0.648–0.747	Reference
SVM (RBF kernel)	0.564	0.474–0.626	Lower AUROC; non-overlapping CI
Random forest	0.694	0.655–0.730	Overlapping CI; difference of 0.005 not clinically meaningful
Gradient boosting	0.678	0.637–0.713	Lower AUROC; Overlapping CI
XGBoost	0.683	0.642–0.717	Lower AUROC; Overlapping CI

AUROC: area under the receiver operating characteristic curve. CI: 95% confidence interval derived from the 2.5th and 97.5th percentiles across 50 cross-validation estimates (5-fold × 10 repeats). No formal significance test was applied; comparisons are based on overlap of confidence intervals and clinical relevance of AUROC differences. All models trained using identical features. Highlighted row = reference model selected for external validation.

**Table 3:** External validation performance for 30-day postoperative mortality prediction (n = 416; 7 deaths).

Model	AUROC (95% CI)	Sensitivity (95% CI) at 90% Specificity	PPV	NPV
GO SOAR full	0.752 (0.570–0.935)	57.1 (14.3–85.7)	10.8	99.2
GO SOAR condensed	0.795 (0.660–0.930)	42.9 (14.3–85.7)	8.8	99.0
SORT	0.769 (0.633–0.905)	14.3 (0–42.9)	2.5	98.4
POSSUM	0.854 (0.720–0.988)	42.9 (14.3–85.7)	7.3	98.9
P-POSSUM	0.822 (0.684–0.959)	42.9 (14.3–85.7)	7.1	98.9
NSQIP	0.686 (0.446–0.927)	42.9 (13.3–85.7)	7.0	98.9

AUROC: area under the receiver operating characteristic curve; PPV: positive predictive value; NPV: negative predictive value. Sensitivity, specificity, PPV and NPV are reported at the prespecified operating point targeting 90% specificity.

**Table 4:** External validation performance for thirty-day postoperative morbidity prediction calculators.

Model	AUROC (95% CI)	Sensitivity (95% CI) at 90% Specificity	PPV	NPV
GO SOAR full	0.694 (0.640–0.757)	30.4 (20.5–41.1)	54.1	77.7
GO SOAR condensed	0.702 (0.645–0.760)	32.1 (22.3–42)	54.5	78.3
POSSUM	0.663 (0.610–0.728)	28.6 (18.2–39.3)	53.1	77.7
NSQIP	0.699 (0.643–0.755)	27.7 (17–38.4)	51.6	77.4

**Table 5:** Calibration statistics—30-day postoperative mortality (n = 416, 7 events).

Model	O/E Ratio (95% CI)	CITL (95% CI)	Calibration Slope (95% CI)	Brier Score
GO SOAR full	1.200 (0.483–2.473)	0.200 (−0.675–0.904)	0.613 (0.089–1.140)	0.0170
GO SOAR condensed	1.167 (0.469–2.405)	0.169 (−0.705–0.869)	0.727 (0.179–1.295)	0.0169
POSSUM	0.154 (0.062–0.317)	−2.156 (−3.018–−1.471)	1.184 (0.525–1.954)	0.0328
P-POSSUM	0.479 (0.193–0.987)	−0.800 (−1.662–−0.114)	0.982 (0.358–1.680)	0.0173
SORT	2.303 (0.926–4.745)	0.862 (0.003–1.543)	0.787 (0.120–1.513)	0.0166
NSQIP	3.334 (1.340–6.868)	1.293 (0.414–2.001)	0.201 (−0.030–0.551)	0.0164

O: observed events; E: sum of predicted probabilities; O/E: observed-to-expected ratio (O/E > 1 = underprediction; O/E < 1 = overprediction). CITL: calibration-in-the-large (0 = perfect mean calibration; 95% CI excluding zero indicates significant mean miscalibration). Calibration slope: 1.0 = perfect; <1 = predictions too extreme; >1 = predictions insufficiently spread. Brier score: lower = better. Highlighted rows = GO SOAR models.

**Table 6:** Calibration statistics—30-day postoperative morbidity (n = 416, 112 events).

Model	O/E Ratio (95% CI)	CITL (95% CI)	Calib. Slope (95% CI)	Brier Score
GO SOAR full	1.018 (0.838–1.224)	0.027 (−0.209–0.256)	0.886 (0.612–1.174)	0.1755
GO SOAR condensed	1.004 (0.827–1.208)	0.006 (−0.229–0.235)	0.936 (0.655–1.232)	0.1740
POSSUM	0.683 (0.562–0.822)	−0.768 (−1.023–−0.52)	0.464 (0.291–0.643)	0.2165
NSQIP	3.025 (2.491–3.640)	1.396 (1.168–1.618)	1.243 (0.849–1.658)	0.2178

O: observed events; E: sum of predicted probabilities; O/E: observed-to-expected ratio (O/E > 1 = underprediction; O/E < 1 = overprediction). CITL: calibration-in-the-large (0 = perfect mean calibration; 95% CI excluding zero indicates significant mean miscalibration). Calibration slope: 1.0 = perfect; <1 = predictions too extreme; >1 = predictions insufficiently spread. Brier score: lower = better. Highlighted rows = GO SOAR models.

## Data Availability

Relevant anonymised data can be obtained on reasonable request from the corresponding author.

## Supplementary Materials

The following supporting information can be downloaded at: https://www.mdpi.com/article/10.3390/cancers18183058/s1, Table S1: GO SOAR Surgical Complexity Score. Table S2: Summary of patient characteristics and events for the external validation cohort. Table S3: Pairwise DeLong comparisons of AUROC for 30-day postoperative mortality prediction. Table S4: Pairwise differences in sensitivity for 30-day postoperative mortality prediction at the operating point targeting 90% specificity. Table S5: Pairwise DeLong comparisons of AUROC for 30-day postoperative morbidity prediction. Table S6: Pairwise differences in sensitivity for 30-day postoperative morbidity prediction at the operating point targeting 90% specificity.
