# Supplementary material for: Predicting the Unpredictable: Development and External Validation of the GO SOAR Surgical Risk Calculator for Data-Driven Predictions of Surgical Complications in Gynaecological Oncology Surgery
Source: Cancers (Basel). 2026 Sep 21;18(18):3058. doi: 10.3390/cancers18183058 (PMC13604734; doi:10.3390/cancers18183058)
Supplement: Supplementary file 1 [file cancers-18-03058-s001.zip › cancers-4463841-supplementary.pdf]

**Table S1**: GO SOAR Surgical Complexity Score

| Pelvic surgery | Score |
|---|---|
| Uterus | 1 |
| Cervix | 1 |
| Fallopian tube(s) | 1 |
| Ovary(s) | 1 |
| Upper vagina | 2 |
| Parametrium | 2 |
| Pelvic peritoneum | 2 |
| Total vagina | 3 |
| Obturator internus | 3 |
| Anus | 3 |
| Vulva | 3 |
| Sciatic nerve | 4 |
| Psoas major | 4 |
| Levator ani | 4 |
| Vulval reconstruction | 4 |
| **Bowel surgery** | |
| Omentum | 2 |
| Abdominal peritoneum | 2 |
| Appendix | 2 |
| Mucous fistula | 2 |
| Large bowel stoma | 2 |
| Small bowel stoma | 2 |
| Rectum | 3 |
| Sigmoid | 3 |
| Ascending colon | 3 |
| Descending colon | 3 |
| Transverse colon | 3 |
| Duodenum | 3 |
| Jejunum | 3 |
| Ileum | 3 |
| Large bowel anastomosis | 4 |
| Small bowel anastomosis | 4 |
| **Urological surgery** | |
| Nephrostomy | 2 |
| Ureteric stents | 2 |
| Bladder | 3 |
| Ureter | 3 |
| Kidney | 3 |
| Urostomy/ileal conduit | 3 |
| Ureteric reimplantation | 4 |
| Trans uretero-ureteric anastomosis | 4 |
| **Upper abdominal surgery** | |
| Spleen | 3 |
| Liver capsule | 3 |
| Gall bladder | 3 |
| Diaphragm resection/stripping | 3 |
| Liver parenchyma | 4 |

| | |
|---|---|
| Stomach | 4 |
| Pancreas | 4 |
| **Lymphadenectomy** | |
| Sentinel | 1 |
| Nodal debulking of enlarged/involved nodes | 2 |
| Systematic pelvic | 3 |
| Systematic para-aortic | 3 |
| Systematic inguinal | 3 |
| Mediastinal | 3 |
| Axillary | 3 |

**Table S2. Summary of patient characteristics and events for the external validation cohort**

| Candidate predictor | Subgroups | No compliations (N=304) | Minor complications Clavien-Dindo I-II (N=78) | Major complications Clavien-Dindo III-V (N=34) |
|---|---|---|---|---|
| **Lost to follow up** | | 0 | 0 | 0 |
| **Age** | Median [IQR] | 63.5 [54.0, 71.0] | 64.0 [56.0, 74.8] | 64.0 [58.0, 73.0] |
| **Ethnicity** | Non-white | 58 (19.1%) | 13 (16.7%) | 8 (23.5%) |
| | White | 246 (80.9%) | 65 (83.3%) | 26 (76.5%) |
| **BMI** | Median [IQR] | 27.6 [23.5, 32.0] | 28.0 [24.6, 33.0] | 31.1 [27.1, 35.3] |
| **ASA grade** | 3, 4 | 70 (23.0%) | 32 (41.0%) | 13 (38.2%) |
| | 1, 2 | 234 (77.0%) | 46 (59.0%) | 21 (61.8%) |
| **ECOG status** | 4, 5 | 1 (0.3%) | 1 (1.3%) | 0 (0.0%) |
| | 1, 2, 3 | 303 (99.7%) | 77 (98.7%) | 34 (100.0%) |
| **Previous laparotomy** | No | 214 (70.4%) | 52 (66.7%) | 16 (47.1%) |
| | Yes | 90 (29.6%) | 26 (33.3%) | 18 (52.9%) |
| **Previous laparoscopy** | No | 237 (78.0%) | 64 (82.1%) | 26 (76.5%) |
| | Yes | 67 (22.0%) | 14 (17.9%) | 8 (23.5%) |
| **Preoperative haemoglobin** | Median [IQR] | 129.5 [119.0, 138.0] | 124.0 [113.2, 136.0] | 123.5 [113.2, 133.8] |
| **Preoperative white cell count** | Median [IQR] | 7.1 [5.6, 8.5] | 7.3 [5.6, 8.8] | 6.8 [5.6, 8.5] |
| **Neoadjuvant chemotherapy** | No | 265 (87.2%) | 59 (75.6%) | 27 (79.4%) |
| | Yes | 39 (12.8%) | 19 (24.4%) | 7 (20.6%) |
| **Surgical modality** | Other | 148 (48.7%) | 57 (73.1%) | 29 (85.3%) |
| | Laparoscopic or robotic | 156 (51.3%) | 21 (26.9%) | 5 (14.7%) |
| **Mechanical bowel preparation** | No | 224 (73.7%) | 55 (70.5%) | 20 (58.8%) |
| | Yes | 80 (26.3%) | 23 (29.5%) | 14 (41.2%) |
| **Intra-operative antibiotics** | No | 50 (16.4%) | 6 (7.7%) | 6 (17.6%) |
| | Yes | 254 (83.6%) | 72 (92.3%) | 28 (82.4%) |
| **FIGO stage** | III, IV | 110 (36.2%) | 38 (48.7%) | 23 (67.6%) |

| | | | | |
|---|---|---|---|---|
| | I, II | 194 (63.8%) | 40 (51.3%) | 11 (32.4%) |
| **Primary cancer** | Cervix | 29 (9.5%) | 11 (14.1%) | 1 (2.9%) |
| | Endometrium | 143 (47.0%) | 21 (26.9%) | 12 (35.3%) |
| | Ovary | 115 (37.8%) | 41 (52.6%) | 20 (58.8%) |
| | Vagina | 0 (0.0%) | 0 (0.0%) | 0 (0.0%) |
| | Vulva | 17 (5.6%) | 5 (6.4%) | 1 (2.9%) |
| **Surgical complexity score** | Low | 234 (77.0%) | 50 (64.1%) | 13 (38.2%) |
| | Moderate | 62 (20.4%) | 19 (24.4%) | 9 (26.5%) |
| | High | 8 (2.6%) | 9 (11.5%) | 12 (35.3%) |

**Table S3. Pairwise DeLong comparisons of AUROC for 30-day postoperative mortality prediction**

| Comparison | ΔAUROC | 95% CI | p-value |
|---|---|---|---|
| GO SOAR full vs GO SOAR condensed | −0.043 | −0.121 to 0.035 | 0.284 |
| GO SOAR full vs SORT | −0.017 | −0.171 to 0.136 | 0.825 |
| GO SOAR full vs POSSUM | −0.103 | −0.353 to 0.148 | 0.421 |
| GO SOAR full vs P-POSSUM | −0.069 | −0.263 to 0.125 | 0.484 |
| GO SOAR full vs NSQIP | +0.065 | 0.000 to 0.130 | 0.049 |

**Note:** ΔAUROC is calculated as GO SOAR full minus the comparator model. Confidence intervals and p-values were obtained using paired DeLong comparisons. P-values are nominal. The nominal p-value of 0.049 for GO SOAR full versus NSQIP did not remain statistically significant after correction for multiple comparisons.

**Table S4. Pairwise differences in sensitivity for 30-day postoperative mortality prediction at the operating point targeting 90% specificity**

| Comparison | 95% CI for difference in sensitivity |
|---|---|
| GO SOAR full − GO SOAR condensed | −12.6% to +50.0% |
| GO SOAR full − SORT | 0.0% to +83.3% |
| GO SOAR full − POSSUM | −57.1% to +80.0% |
| GO SOAR full − P-POSSUM | −33.3% to +50.0% |
| GO SOAR full − NSQIP | −16.7% to +57.1% |

**Note:** Differences are calculated as the sensitivity of GO SOAR full minus the sensitivity of the comparator model. The 95% confidence intervals were obtained using paired bootstrap resampling with 2,000 repetitions. All confidence intervals included zero, indicating substantial uncertainty around the observed between-model differences.

**Table S5. Pairwise DeLong comparisons of AUROC for 30-day postoperative morbidity prediction**

| Comparison | ΔAUROC | 95% CI | p-value |
|---|---|---|---|
| GO SOAR full vs GO SOAR condensed | −0.004 | −0.010 to +0.001 | 0.127 |
| GO SOAR full vs POSSUM | +0.029 | −0.026 to +0.084 | 0.305 |
| GO SOAR full vs NSQIP | −0.001 | −0.058 to +0.056 | 0.980 |

**Note:** ΔAUROC is calculated as GO SOAR full minus the comparator model. Confidence intervals and p-values were obtained using paired DeLong comparisons. None of the pairwise AUROC differences reached statistical significance.

**Table S6. Pairwise differences in sensitivity for 30-day postoperative morbidity prediction at the operating point targeting 90% specificity**

| Comparison | 95% CI for difference in sensitivity |
|---|---|
| GO SOAR full − GO SOAR condensed | −6.7% to +3.5% |
| GO SOAR full − POSSUM | −10.2% to +14.5% |
| GO SOAR full − NSQIP | −9.6% to +19.1% |

**Note:** Differences are calculated as the sensitivity of GO SOAR full minus the sensitivity of the comparator model at the operating point targeting 90% specificity. The 95% confidence intervals were obtained using paired bootstrap resampling with 2,000 repetitions. All three confidence intervals include zero, indicating substantial uncertainty around the observed between-model differences in sensitivity.
